# Hybrid Material Based on an Amorphous-Carbon Matrix and ZnO/Zn for the Solar Photocatalytic Degradation of Basic Blue 41

**DOI:** 10.3390/molecules25010096

**Published:** 2019-12-26

**Authors:** Silvania Lanfredi, Marcos A. L. Nobre, Po S. Poon, Juan Matos

**Affiliations:** 1 Department of Chemistry and Biochemistry, São Paulo State University (Unesp), School of Technology and Sciences, Laboratory of Composites and Ceramics Functional, Presidente Prudente 19000-000, Brazil; 2 Department of Physics, São Paulo State University (Unesp), School of Technology and Sciences, Presidente Prudente 19000-000, Brazil; marcos.nobre@unesp.br; 3 Technological Development Unit (UDT), University of Concepcion, Concepcion 4070386, Chile; p.poon@udt.cl; 4 Millennium Nuclei on Catalytic Processes towards Sustainable Chemistry (CSC), Santiago 7820436, Chile

**Keywords:** carbon/ZnO composites, basic blue 41, photocatalytic degradation, solar irradiation, lixiviation, amorphous carbon

## Abstract

Innovative composites based on an amorphous-carbon matrix containing a second phase ZnO oxide and/or highly dispersed Zn metallic were synthesized via a modified Pechini route, in which a partial pyrolysis method was reached. Studies of adsorption in the dark and the photocatalytic activity for the cationic azo-dye, basic blue 41, and degradation were carried out. X-ray diffraction patterns for the carbon matrix and its composite with Zn show characteristics of the amorphous carbon. The infrared in the mid region of the composite prepared with ZnO and Zn exhibit vibrational bands related to bonds zinc oxide. The surface pH of the material is the main factor responsible for the adsorption of the azo-dye, but the contribution of mesopores favored the diffusion of molecules from the bulk of solution to the pore framework. Esters-like functional groups on the surface of carbons hinder the adsorption of the azo-dye. When Zn is embedded within amorphous carbon the photocatalytic activity of the composites showed up to 2.4 higher than neat ZnO. The enhancement in the photocatalytic activity and stability of C/ZnO/Zn and C/Zn composites is discussed in terms of a protector effect by the carbon layers inserted in composites. Carbon layers are responsible to inhibit the lixiviation of ZnO particles along irradiation.

## 1. Introduction

The environmental pollution is a global problem with a strong impact on the human health [1] and food security [2]. One of the major issues is the water pollution [3,4] by residual industrial liquids (RILs). Thus, a wide range of methodologies such as adsorption [5,6], biodegradation [4,7], and the so-called advanced oxidation processes (AOPs) [8,9,10,11,12,13,14,15,16,17,18,19,20,21,22,23,24] have been applied for the treatment of polluted water. Heterogeneous photocatalysis is one of the most efficient AOPs [9,11,12,14,15,16]. This technology uses metal oxide-based semiconductors such as TiO_2_ [9,11,12,17,18], ZnO [18,19,20,21], SnO_2_, [22] and CeO_2_ [23] among others. It has been reported that TiO_2_ is the best photocatalyst to mineralize a wide range of pollutants to carbon dioxide and water [14,24], and this feature has been addressed as an exceptional combination of electronic structure, light absorption properties, charge transport characteristics, and low cost. However, TiO_2_ has some important scaling-up limitations [25] such as the low activity under visible light, the high recombination rate of photogenerated electron–hole pairs, and a low stability in terms of their recovery and reutilization. In addition, TiO_2_ has been recently listed as a suspected carcinogen to humans [26]. Thus, improvement of the performance of photocatalysts is required for the treatment of polluted water. In this sense, ZnO has received increasing attention for the photocatalytic remediation of polluted water [18,19,20,21,27,28]. ZnO is a n-type semiconductor material with a high thermal conductivity, wide band gap (3.37 eV), that is able to generate charge carriers when stimulated with UVA light, biocompatible, and with a high natural abundance, and low-cost. However, it has been reported [29,30] that ZnO is lixiviated in aqueous phase reactions even without UV-irradiation. The inhibition process of lixiviation of ZnO under UV-visible irradiation has been reported for carbon-based materials [18,27,28] and carbon quantum dots as dopants [31]. In major part, this effect occurs due to the high reduction potential of carbon materials [32,33,34,35], which allows an efficient electron-donor transference from the graphene-based layers to the semiconductor, protecting them from thermo- or photo-oxidation processes, even at strong oxidation conditions such as UV irradiation and high temperature. The particle size, morphology, surface area, crystalline framework, and composition are responsible of the optoelectronic properties of ZnO [18,19,20,21,27,28,29,30,31] including the photocatalytic activity. Properties of ZnO-based materials are highly dependent of the synthesis method [18,19,20,21,27,28,29,30,31,36,37,38,39,40,41,42] including viscosity of slurry suspensions [18,20,27], Pechini synthesis [28,42], polymeric precursor degradation [29], sol-gel following annealing [19], microwave-assisted solvothermal synthesis [31], high-energy ball-milling [36], spray pyrolysis [37], suction casting [39], and hydrothermal synthesis [21,38,40,41], among others. These set of works have shown that ZnO is a versatile ceramic material, which can be used in several applications such as solar cells [31], varistors [36], luminescent materials [37], and photocatalysts [18,19,20,21,27,28]. An interesting approach is to combine ZnO with carbon-based materials [18,20,27,28,40,41] because these hybrid composites have shown a higher stability and photocatalytic activity than that observed on neat ZnO. The basic blue 41 has been chosen as target azo-dye because is widely used in the textile industry [43,44], and due to the growing concerning about the carcinogenic and mutagenic effects of this azo-dye [44].

The present work reports an ecofriendly method for the synthesis of C/ZnO/Zn hybrid materials and their evaluation in the photocatalytic degradation of the cationic azo-dye basic blue 41 (BB41). The kinetics of photodegradation of the azo-dye was followed under artificial solar irradiation to verify the activity and stability of composites. The model of adsorption is discussed.

## 2. Results and Discussion

### 2.1. Characterization of Samples

**Figure 1:**
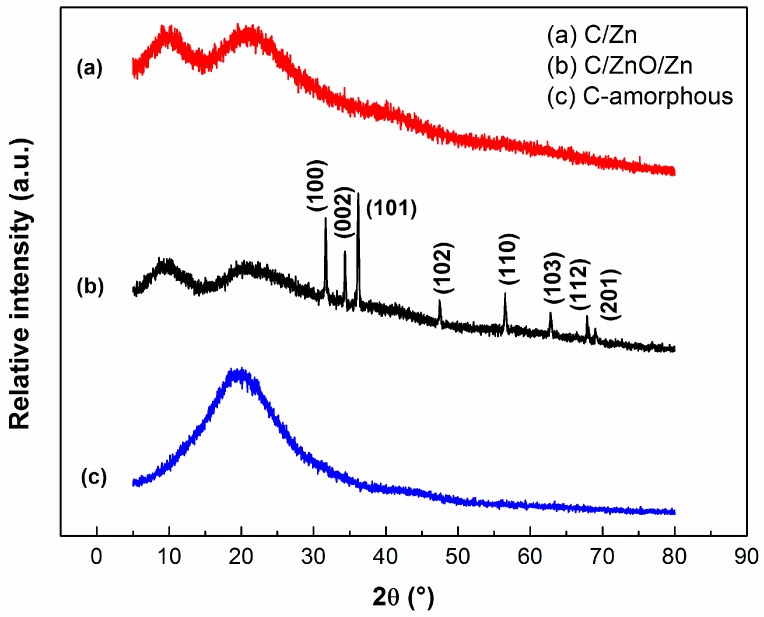
XRD patterns of the samples. (**a**) C/Zn; (**b**) C/ZnO/Zn; (**c**) C-amorphous.

Figure 1 shows the XRD patterns of the amorphous carbon, C/Zn, and C/ZnO/Zn composite. The XRD patterns for the C/Zn composite (Figure 1a), and the amorphous carbon matrix (Figure 1c) did not exhibit diffraction lines indicating a low crystallinity or high dispersion avoiding metallic clustering. Even more, it can be suggested that the diffraction lines of Zn-containing phases can be superposed with those from the amorphous carbon matrix (Figure 1c). 

Counterclockwise, the diffraction lines at 2θ = 31.78°, 34.47°, 36.26°, 47.70°, 56.60°, 62.81°, 67.93°, and 69.39° observed in the Figure 1b can be indexed to the Bragg reflections (100), (002), (101), (102), (110), (103), (112), and (201), respectively, for the crystallographic planes of a zincite crystal structure [45] of hexagonal symmetry and space group P63mc (# 186) (JCPDS No. 36-1451). The Figure S1a (Supplementary Material) shows that the diffraction lines of the commercial ZnO are similar to those observed in the Figure 1b, concluding the present methodology did not affected the crystallographic framework of ZnO. 

**Figure 2:**
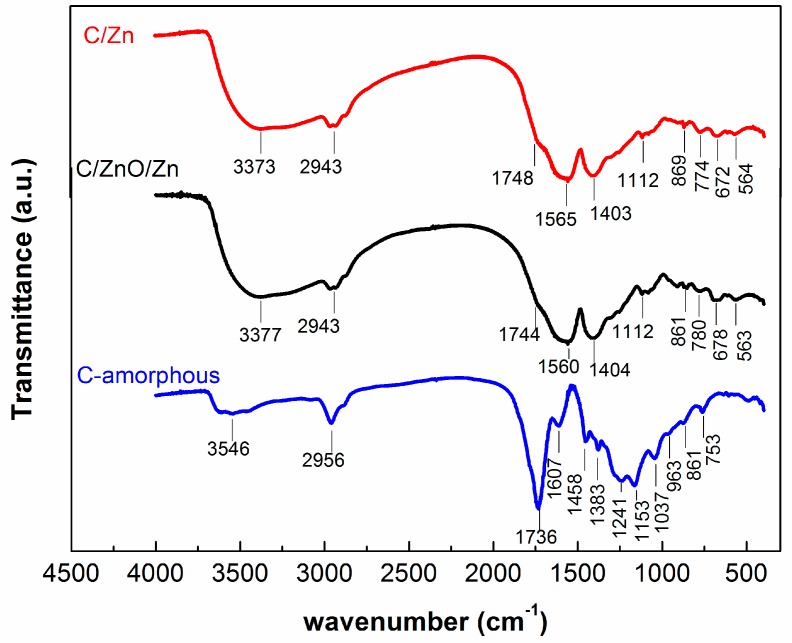
FTIR spectra of C/Zn, C/ZnO/Zn, and C-amorphous.

The set of analysis of chemical bonds of samples were carried out by FTIR transmittance spectra as shown in Figure 2. For the sake of comparison, the FTIR spectra for the commercial ZnO is shown in the Figure S1b (Supplementary Material). Table 1 lists the assignment of the absorption bands for the FTIR spectra of the Figure 2. 

Absorption bands in the range between 3373–3546 cm^−1^ are associated to the symmetrical stretching of hydroxyl groups (OH^−^) [46,47], while the band at 2943 cm^−1^ can be assigned to the C–H stretching [27,28]. Both group bond deformation of the OH and C–H are positioned between 1607–1403 cm^−1^ [28,48], respectively. Figure 2 also shows vibrational bands associated with the C=O stretching between 1748–1736 cm^−1^, and to the C–O stretching (1383–1037 cm^−1^) of esters group that correspond to the polyester formed during the polyesterification process [29,49,50]. Bands positioned in the region between 963–753 cm^−1^ has being assigned to the C–C stretching [27,28]. 

Absorption bands positioned in the range between 672–563 cm^−1^, picked up in the C/Zn and C/ZnO/Zn samples can be assigned to the metal-oxygen bonds of Zn–O [45,51,52]. This assignment has been confirmed from the FTIR spectra for the commercial ZnO in Figure S1b (Supplementary Material). Two strong vibrational bands at 431 and 523 cm^−1^ can be assigned to the stretching modes of ZnO bond [45], while the band observed at 871 cm^−1^ can be assigned to stretching modes of ZnO bond in tetrahedral coordination [53]. The absorption band centered at 1543 cm^−1^ can be assigned to chemisorbed water molecules on ZnO surfaces [45], and to the O-H stretching mode at 3356 cm^−1^. It can be concluded that most of the functional groups detected by FTIR are chemical groups presented in the surface of the amorphous carbons as can be seen after comparison of the spectra. 

**Figure 3:**
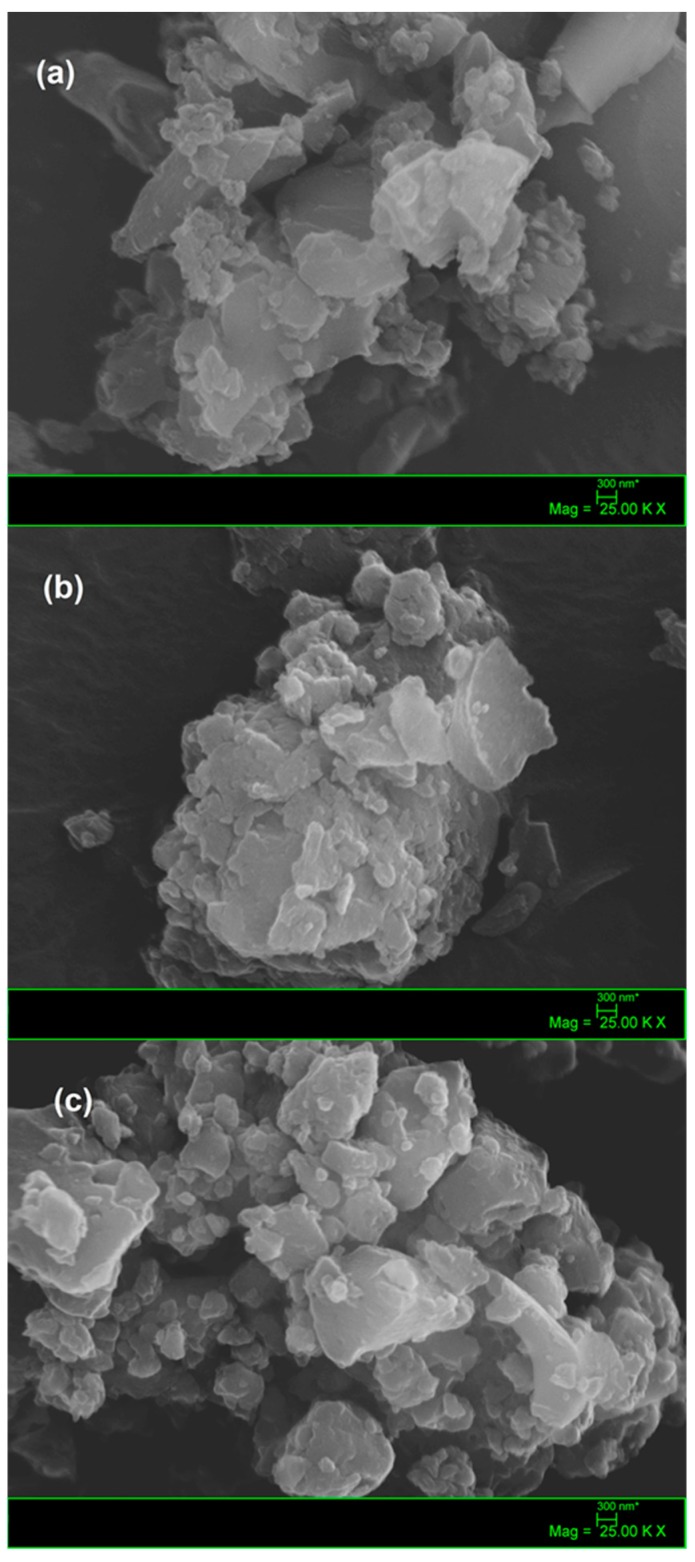
SEM images. (**a**) C-amorphous; (**b**) C/Zn; (**c**) C/ZnO/Zn.

The morphology of composites and the amorphous carbon was verified by scanning electron microscopy SEM showed in Figure 3. SEM image for the C-amorphous (Figure 3a) showed the presence of clusters of different shapes and sizes, constituting a matrix with high carbon content and organized in the shape of overlapping plates. 

It can be suggested that the smallest carbon particles were formed by the fragmentation during the slow-pyrolysis step of the polymeric gel described above. A similar fragmentation has been observed by Liu and coworkers [54], who have reported a close mechanical behavior of the non-uniform porous char particles during the combustion of pulverized coal. For the C/Zn composite (Figure 3b) nanostructures in the form of plates were also observed, however metallic nanoparticles were not observed in this sample, compatible with high dispersion level of Zn in the material. This result agrees with the amorphous character XRD pattern observed for this sample (Figure 1a), suggesting that Zn-containing nanoparticles should be embedded within the carbon matrix, as reported by Chae and coworkers [55], for a nanosized zinc oxide electrode in carbon-based lithium-ion batteries. On the contrary, the SEM image of the C/ZnO/Zn composite (Figure 3c) shows the presence both of large particles agglomerated in the form of plates ascribed to amorphous carbon and crystalline structures of smaller size ascribed to clusters of ZnO nanoparticles dispersed on the larger structures. This aspect is in agreement with a more crystalline structure observed in the XRD pattern discussed above (Figure 1b). As a general fact, it can be highlighted no evidence of porosity in the samples (not even macro or mesoporosity) can be observed from the SEM images.

**Figure 4:**
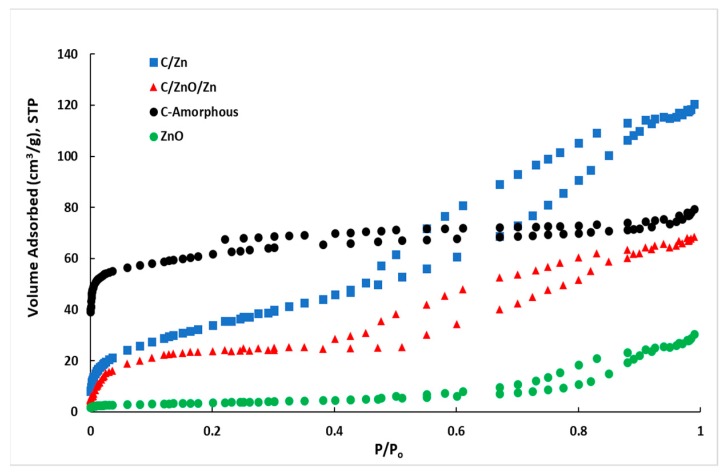
Adsorption–desorption isotherms of N_2_ at −196 °C for the different samples.

Figure 4 show the N_2_ adsorption-desorption isotherms at −196 °C and Table 2 contains a list of the textural properties estimated from the isotherm’s hysteresis shape. For the C-amorphous sample a classical type-I adsorption isotherm can be observed, indicating C-amorphous is a predominantly microporous material with a small contribution of mesopores to the total volume of pores. However, the hysteresis loop (H4) for this sample does not close (not even at P/P_0_ = 0.2), which is characteristic of materials with constricted micropores [56].

On the contrary, all the other adsorption isotherms are type IV with a H2(b) hysteresis loop [56], indicating that the present materials are mainly composed by a mesoporous framework with a small-to-moderate contribution of micropores. However, the hysteresis loop for the neat semiconductor (ZnO) is different from that observed in the composites in presence of amorphous carbon, where it showed a closing desorption branch at ca. 0.4 relative pressure while it close at ca. 0.6 for ZnO.

The hysteresis classification suggests that with except than ZnO, both C/Zn and C/ZnO/Zn composites contain both micropores and small mesopores [56] as suggested by the representative values observed in Table 2. In presence of Zn or ZnO, the composites C/Zn and C/ZnO/Zn decreased remarkably the surface area in comparison than C-amorphous sample. In other words, the crystalline phase composed by ZnO and few Zn nanoparticles are responsible of the blocking of pores—mainly micropores—formed in the amorphous carbon. This can be inferred from the decrease in the ratio V_DR_/V_0.99_ from 66% for C-amorphous down to 26% and 19% for C/Zn and C/ZnO/Zn, respectively. Values listed in Table 2 suggest that neat ZnO is 98% mesoporous, however, this sample is highly crystalline, as can be seen from Figure S1a (Supplementary Material) and therefore, the mesoporosity in this sample is due to interparticle mesopore voids [25].

For the sake of comparison, the volume of pores for diameters lower than 1 nm (V_<1 nm-DFT_) were also estimated from density functional theory (DFT) [57,58]. It can be seen that the contribution of supermicropores to the volume of pores estimated by DFT is ca. 32% for the amorphous carbon while is only 7% and 5% for C/Zn and C/ZnO/Zn, indicating that micropores are the more important fraction of pores that are being blocked by Zn nanoparticles. It is expected that the contribution of micro and mesopores influence the kinetics of adsorption of the BB41. These studies are discussed as follows.

### 2.2. Influence of Surface pH Upon the Kinetics of Adsorption in the Dark

**Figure 5:**
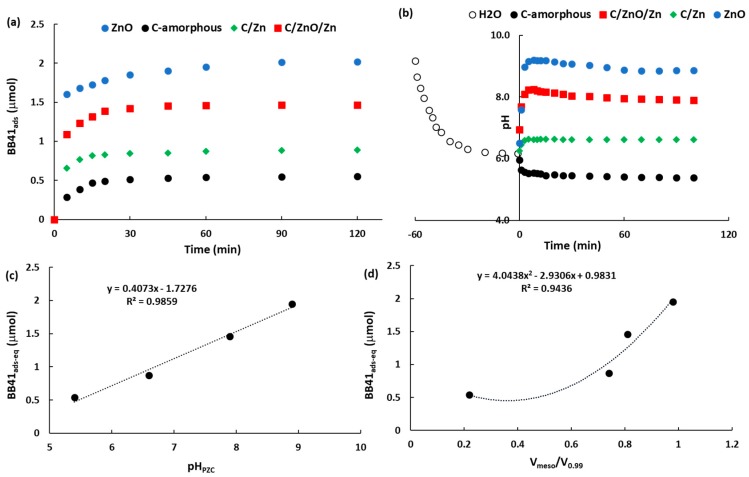
(**a**) Kinetics of BB41 adsorption in the dark; (**b**) pH changes as a function of time; (**c**) relationship between BB41 adsorbed and the pH_PZC_; (**d**) relationship between BB41 adsorbed and the ratio V_meso_/V_0.99_.

The Figure 5a shows the kinetics of BB41 adsorption in the dark on the present samples. All the samples showed that the kinetics of BB41 adsorption has achieved the equilibrium condition after 60 min. Thus, this time will be used before the irradiation in the photocatalytic tests. Table 3 shows a summary of the BB41 adsorbed after 60 min as well as the surface pH (pH_PZC_) obtained from the plots of Figure 5b according to the drift-pH method [25].

An excellent linear correlation is observed in the Figure 5c where the higher the surface pH of the adsorbent the higher the adsorption of BB41 at equilibrium condition. This result agrees with the cationic nature of the dye and with results reported elsewhere from different research groups [59,60,61], whom showed that the adsorption of BB41 on brick waste [59], zeolites [60], and activated carbon [61] is highly influenced by the pH of solution, as well as the functional groups on the surface of the adsorbents. In other words, the surface pH of the adsorbent is responsible of the thermodynamic trend to adsorb the cationic BB41 azo-dye. 

For the loading of adsorbent used in the present work (ca. 0.1 g L^−1^), the BB41_ads-eq_ values reported in the Table 3 correspond to 2.6 mg g^−1^, 4.2 mg g^−1^, 7.0 mg g^−1^, and 9.4 mg g^−1^ adsorbed on C-amorphous, C/Zn, C/ZnO/Zn, and ZnO, respectively. These values are one order magnitude lower than those reported for the BB41 adsorption on brick waste (60–70 mg g^−1^) [59], on zeolites (ca. 190 mg g^−1^) [60], and on activated carbon (125 mg g^−1^) [61]. This result was expected since these works were conducted at one order magnitude higher loading of adsorbents (ca. 1 up to 2.5 g L^−1^), and using higher concentration of BB41, up to 200 ppm, instead 12.5 ppm as in the present work.

In addition, Figure 5d shows a 2-degree polynomial correlation between the quantity adsorbed of BB41 at equilibrium condition, and the ratio between the volume of mesopores and the total pore of volume (V_meso_/V_0.99_). Based on the van der Waals radii, it can be estimated that the molecular area (σ_BB41_) of the cationic form of the BB41 molecule (Figure S2, Supplementary Material) is ca. 2.45 nm^2^. This value suggests that not only high loading, but also an important quantity of mesopores is required to guarantee an efficient diffusion from the bulk of solution to the porous framework of the adsorbent. In other words, despite of the C-amorphous has the highest surface area (Table 2), this sample contains the lowest contribution of the volume of mesopores to the total pore volume, and thus, the diffusion of BB41 molecules can be hindered by the acidic functional groups on its surface, since this sample showed the lowest surface pH (Table 3). This suggestion is confirmed from the experimental estimation of the σ_BB41_ using the value for the BB41 adsorbed at equilibrium condition (Table 3). For the case of the amorphous carbon sample σ_BB41_ was ca. 60 nm^2^ molecule^−1^, which is clearly higher than the theoretic value (2.45 nm^2^ molecule^−1^). On the contrary, the σ_BB41_ value estimated for ZnO, C/ZnO/Zn, and C/Zn are ca. 1.11 nm^2^ molecule^−1^, 8.53 nm^2^ molecule^−1^, and 23.8 nm^2^ molecule^−1^, which are clearly lower than that observed on the C-amorphous, suggesting that the functional acidic groups on the surface of the amorphous carbon are responsible to hinder the approach of BBV1 molecules. Thus, for a better understanding of the influence of the surface pH and pore size distribution of the samples, a carefully study of BB41 adsorption was performed by using the pseudo-first order, pseudo-second order and the intraparticle diffusion (IPD) kinetic models described in the Table S1 (Supplementary Material). Table 3 lists the set of kinetic results. 

From comparison between values for the pseudo-first order and pseudo-second order reported in Table 3, it can be point out that ZnO showed a better adjustment to a chemisorption model because R_k2_ is much higher than R_k1_. Otherwise, the C-amorphous sample followed a pseudo-first order kinetic model (R_k1_ < 0.95). For the composites, from the similar features observed in the values of the regression factors, it seems to be that a combination of physisorption and chemisorption is favored on C/Zn and C/ZnO/Zn composites. These analyses confirmed that the adsorption of BB41 is mainly controlled by the chemisorption promoted by the electrostatic attraction between the negative charged surface of Zn-containing samples and the cationic dye molecules, in accordance with contributions reported by Zarezadeh-Mehrizi and Badiei [62] for the adsorption of BB41 on negatively charged nanoporous silica. As a matter of fact, these authors reported values for k_1_ and k_2_ in the same order magnitude than the results reported in the present work.

It can be seen from the plots of the intraparticle diffusion model (IPD) for the C-amorphous, the C/Zn and C/ZnO/Zn samples in Figures S3c, S4c and S5c (Supplementary), respectively, that two steps are involved in the BB41 adsorption, described by the external mass transfer (boundary-layer diffusion) and intraparticle diffusion [62,63]. Thus, the plot suggests that the initial curved part of the plot indicates a boundary-layer effect, while the second linear portion is due to intraparticle or pore diffusion. This behavior’ is not observed for the ZnO sample where only a perfect linear plot is observed (Figure S6c, Supplementary), indicating an important boundary-layer effect due to the surface diffusion in agreement with the highest value for the C constant (Table 3), as suggested by the correlation between the values for C constant and pH_PZC_ in Table 3.

In other words, the boundary-layer diffusion for the adsorption of BB41 is mainly controlled by the electrostatic attraction, with additional contribution of meso and micropores stemming the intraparticle or pore diffusion step, as reported by Lee and coworkers [63] elsewhere. It seems to be evident that both phenomena of adsorption would influence the photocatalytic degradation of BBV1 as discussed as follows.

### 2.3. Photocatalytic Tests

**Figure 6:**
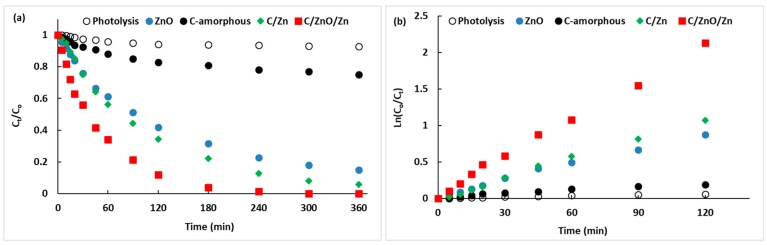
(**a**) Kinetic of BB41 photodegradation under artificial solar irradiation; (**b**) linear regression of the kinetic data from Figure 6a.

Figure 6 shows the kinetics of photocatalytic degradation of BB41 and the linear regression of the kinetic data. A summary of the kinetic results is listed in Table 4. As discussed previously, the data was normalized to the concentration of BB41 in solution after 60 min of adsorption in the dark. For the sake of comparison, direct photolysis has been included and in absence of solids, the conversion of BB41 was only 7% after 6 h irradiation, indicating that direct photolysis is negligible in the present experimental condition using artificial solar irradiation, where the UV component is only 6% of the total radiant flow. It means that the present results of BB41 photoconversion are due to the photocatalytic activity of the samples.

The amorphous carbon is a photochemically active material showing a BB41 conversion of 25% after 6 h irradiation. This level of conversion was expected since some functional groups presented in the surface of the amorphous carbon are photoactive as shown by our group [25,28,64,65,66], and by other groups [67,68,69,70,71]. In the present case, the photoactivity in the amorphous carbon can be ascribed to the polyester groups observed in the Figure 2 and summarized in Table 1. However, this low photoactivity is ascribed to a low thermodynamic trend to adsorb the cationic BB41, since this carbon is characterized by an acidic surface. In other words, in the case of the C-amorphous, the limiting-step seems to be the adsorption of the pollutant, as suggested by the low C value (Table 3), according to the IPD model.

Figure 6a shows that the composites C/ZnO/Zn and C/Zn are more photoactive that the neat ZnO, even despite of the later adsorbed more BB41 at dark conditions (Table 3). After 3 h irradiation, the BB41 conversion was 96%, 78%, and 69% (Table 4) on C/ZnO/Zn, C/Zn, and ZnO, respectively. Despite of ZnO showed an initial photocatalytic activity higher than C/Zn, however, after 45 min irradiation, it is clear from Figure 6a that ZnO was monotonically inhibited along the irradiation, which is attributed to the lixiviation of ZnO nanoparticles. This detrimental effect has been also reported by other authors [30,72,73], and it is the consequence of the extensive protonation of the hydroxy groups of ZnO which is favored at acid pH. On the contrary, the two C-containing composites showed a better stability along reaction, and for the C/ZnO/Zn sample, almost 100% BB41 photodegradation was observed after only 3 h irradiation. The higher photoactivity and stability of this sample is ascribed to the protector effect of carbon [32,33] from the lixiviation phenomena, due to carbon-based materials maintain a reductive chemical environment, inhibiting the lixiviation of Zn-containing nanoparticles by the oxidative acid attack.

The linear regression showed in Figure 6b were used to estimate the first-order apparent rate-constants (k_app_). It can be seen the Zn-containing composites showed regression factor (R_kapp_) higher than 0.99 and thus, it can be concluded that the BB41 photocatalytic degradation follows a first-order mechanism, which is also observed for the C-amorphous and the direct photolysis with regression factors ca. 0.96. It must be point out that the k_app_ values for C/Zn and C/ZnO/Zn samples, ca. 9.2 × 10^−3^ min^−1^ and 17.2 × 10^−3^ min^−1^, respectively, are higher or in the same magnitude order that values reported elsewhere which are in the range 2–40 × 10^−3^ min^−1^ for more complex photocatalysts [74,75,76,77,78] than the reported in the present work. The comparison between samples can be easily obtained from the ratio k_app-i_/k_app-ZnO_ since it allows further verification of the enhancement in the photocatalytic activity of a sample relative to that observed on the neat ZnO. It can be seen in Table 4 that C/ZnO/Zn and C/Zn are 2.4 and 1.3 times higher photoactive than ZnO alone, even when the Zn-content in the composites is ca. 50 wt. %. In other words, there is further evidence that the amorphous carbon and the ZnO catalysts presented an interaction factor between both solids that estimated from the ratio [k_app-C/ZnO/Zn_/k_app-ZnO + kapp-C-amorphous_] corresponds to a synergy effect of ca. 1.9. This phenomenon has been also reported by Stathatos and coworkers [76] using a TiO_2_/palygorskite composite nanocrystalline films for the photodegradation of basic blue 41. Thus, despite on the surface functional groups on the C-amorphous hindered the BB41, the protector effect of the amorphous carbon under UV-visible irradiation is responsible of an enhancement of the photocatalytic activity of ZnO by a factor of 2.4. 

**Figure 7:**
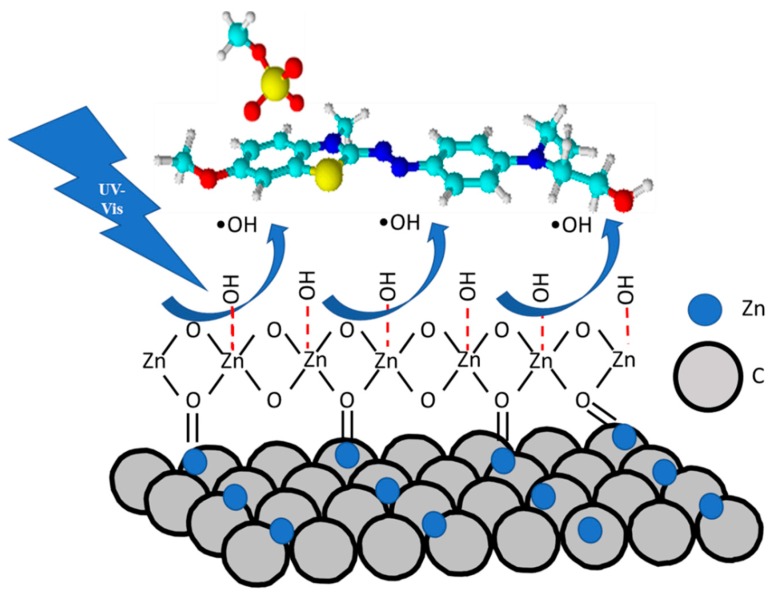
Mechanism for the photocatalytic degradation of the BB41 on C/Zn and C/ZnO/Zn composites.

A schematic mechanism for the photocatalytic degradation of the BB41 on the present C/Zn and C/ZnO/Zn composites is shown in the Figure 7. This mechanism shows at first place that carbonyl groups on the surface of carbon materials can be coordinated to the ZnO nanoparticles to form a well stable composite, explaining the inhibition effect in the lixiviation of ZnO by means of the protection of carbon layers. Secondly, it can be seen that the hydroxy groups adsorbed on the ZnO are transformed to hydroxy radicals (OH) under UV-vis irradiation. These radical oxygen species are responsible of the photocatalytic activity for the degradation of BB41.

Further studies of long-term stability based on the consecutive photocatalytic runs and with other types of carbon-based materials are being investigated for a better understanding of this protective phenomenon. 

### 2.4. General Discussion

For a better understanding of the photocatalytic results, some additional characterization of the materials such as elemental composition and diffuse reflectance UV-VIS spectroscopy (DR/UV-VIS) was included. Table 5 contains a summary of the composition of the photocatalysts.

As expected, amorphous carbon is characterized by a high proportion of C which is decreased remarkably when Zn is incorporated to the composite. It is interesting to remark that the H and organic O contents in these samples remains without variations while the C content decreased for the C/ZnO/Zn composite in comparison to C/Zn sample. This result agrees with the higher surface area observed for C/Zn composite as discussed above (Table 2). In addition, the content in Zn and inorganic O increased in the C/ZnO/Zn composite in comparison to C/Zn sample (44 % against 37 %). This increase was ca. 1.2 times higher which is close to the nominal expected increase (ca. 1.5). Thus, it can be concluded that the present methodology is useful for the linkage of metallic zinc nanoparticles within the amorphous carbon structure. Thus, from the data in Table 5 and the stoichiometric consideration for the ZnO formula, it can be estimated that in 0.1 g L^−1^ of photocatalysts loading, ca. 0.035 g L^−1^ and ca. 0.030 g L^−1^ correspond to Zn composition in the C/ZnO/Zn and C/ZnO photocatalysts, respectively. We do believe this difference is not representative enough to be responsible of the variations in the photocatalytic activity. Indeed, the enhancement in Zn content is only 1.2 times higher in C/ZnO/Zn in comparison to C/ZnO photocatalysts, while the photocatalytic activity was ca. 2 times higher (Table 4). Thus, additional analysis was performed and discussed as follows.

**Figure 8:**
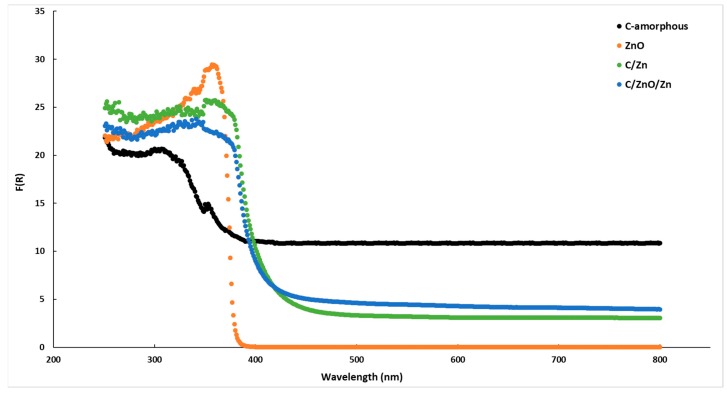
DR/UV-VIS spectra of the samples.

The Figure 8 shows the DR/UV-VIS spectra for the present samples, including the commercial ZnO and the amorphous carbon. It can be seen that amorphous carbon presents an important absorption of photons in all the range of the spectra. This result agrees with the black-body-like behavior of the carbon-based materials. On the other hand, commercial ZnO shows the expected trend for this semiconductor showing an almost vertical decrease after achieved the maximum in the absorption band. The energy band gap estimated for ZnO using the Kubelka-Munk function [25] was ca. 3.23 eV which is in good agreement with previous reports [18]. By contrast, the Zn-containing samples showed a much less decrease in the absorption band, and it is clear that these samples presented a red-shift. Accordingly, the energy band gaps estimated for these samples were ca. 2.97 eV and 2.93 eV for C/ZnO/Zn and C/Zn, respectively. Thus, the higher the carbon content the lower the energy band suggesting that doping of the ZnO crystalline framework by C atoms is an important parameter to be considered. However, in spite of the energy band gap for C/Zn sample is lower than that of C/ZnO/Zn composite, the photocatalytic activity was clearly lower (Table 4). Thus, as discussed above, it can be concluded that for the specific case of BB41, the surface pH seems (Table 3) to be the most important role affecting the photocatalytic activity, and thus, a faster process of adsorption of the pollutant would be the driven-force for the enhancement in the catalytic activity.

In addition, to highlight the potential of the present nanostructured materials, the best photocatalyst (C/ZnO/Zn) was selected for a study of consecutive photocatalytic runs (reuse), together with an analysis by atomic absorption spectroscopy of the Zn lixiviated to the solution during reaction. For the study of reuse of catalysts, after 3h of reaction when BB41 conversion achieved ca. 100% using the C/ZnO/Zn catalysts (Figure 6a), a concentrated aliquot of BB41 was added to the reactor to maintain the same initial concentration (12.5 ppm) and same volume (1 L) at the end of each run. After this, a period of 60 min adsorption in the dark was performed and then, irradiation was started again for the next catalytic run.

**Figure 9:**
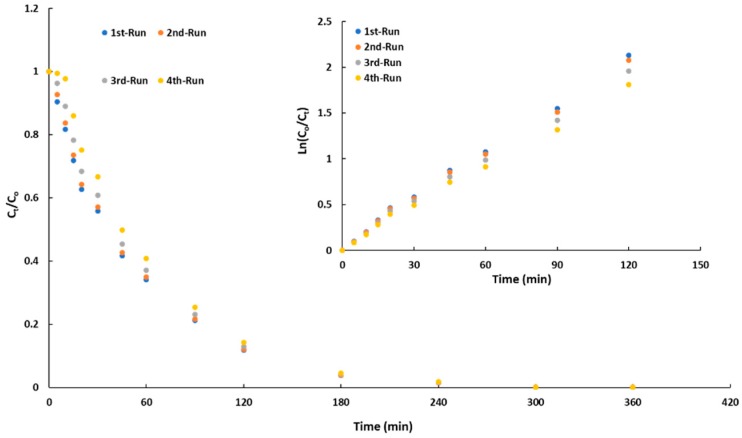
Kinetics of BB41 photodegradation at different photocatalytic consecutive runs. The figure inset contains the linear regression of the kinetic data.

Figure 9 shows the kinetics of BB41 photodegradation at consecutive photocatalytic runs while the figure inset contains the linear regression of the kinetic data. Table 6 lists a summary of the kinetics results and the data of Zn leached along these catalytic runs.

It can be seen that the photocatalytic activity was not significantly affected during four consecutive cycles. Though the first-order apparent rate-constants (Table 6) slightly decrease with the increase in the numbers of cycles, Figure 9 shows that after 180 min, more than 90% of BB41 was degraded in all the cycles concluding that even after four consecutive photocatalytic runs, C/ZnO/Zn is much more photoactive than the commercial pristine ZnO in only one cycle. This potential photocatalytic activity has been ascribed to a high stabilization of metallic zinc nanoparticles within the carbonaceous matrix. As discussed aboved, carbon support would protect the Zn particles from the lixiviation effect. The data listed in the Table 6 indicated that even after four consecutive photocatalytic runs, only 3.4% of Zn was leached to the solution in agreement with the high photocatalytic activity observed along the four cycles of BB41 photodegradation.

It is well-known that the electron affinity of Zn is ca. 0 kJ/mol. Accordingly, metallic Zn is an efficient electron donor component which is able to inject hot electrons to the conduction band of ZnO, and thus, promoting the formation of super oxo anion radicals (O_2_•^−^). In short, the composite C/ZnO/Zn play the role of a Z-type photocatalysts and thus, metallic Zn nanoparticles contributes to the enhancement in the activity of the whole photocatalysts.

## 3. Experimental

### 3.1. Synthesis

Citric acid (C_6_H_8_O_7_H_2_O), ethylene glycol (HOCH_2_CH_2_OH), zinc nitrate [Zn(NO_3_)_2_·6H_2_O)] and zinc oxide (ZnO) were high purity reagents (>98%, Sigma-Aldrich, San Luis, MO, USA). The cationic azo-dye basic blue 41 C_2_0H_26_N_4_O_6_S_2_ here denoted BB41 was also purchased from Sigma-Aldrich (purity ca. 40%). The hybrid materials synthesized are composed by zinc oxide and/or metallic zinc dispersed in a matrix of amorphous carbon. Samples were prepared by a sol-gel modification of the Pechini method [42,79] following by a partial pyrolysis method reported by our group for the synthesis of Ni-doped niobate/carbon composite [48]. In a typical synthesis, a polymeric resin is produced by the esterification reaction between citric acid as the metal chelate complex and ethylene glycol as the polyhydroxy alcohol. Citric acid was dissolved in ethylene glycol (mass ratio of 40:60) with continuous magnetic stirring at 70 °C, to promote the polymerization. After the polymeric gel was obtained the Zn precursors were added gradually. Two different materials were produced by adding Zn(NO_3_)_2_.6H_2_O in absence or presence of ZnO. In this case, ZnO was firstly added and stabilized by vigorous stirring by 60 min. In this composite, the molar ratio ZnO/Zn was 0.5:1.0 and the molar ratio citric acid/metallic cation was ca. 2:1. The as prepared materials were submitted to calcination in an ceramic oven under static air atmosphere by one-step calcination heating cycle from room temperature up to 300 °C at constant heating rate ca. 1 °C min^−1^ and then by 2 h at this temperature. Composites were deagglomerate in the agate mortar and sieved (350-mesh). Samples were denoted as C-amorphous (in absence of Zn), C/Zn (in absence of ZnO) and C/ZnO/Zn, when both Zn sources (oxide and nitrate) were added to the polymeric gel.

### 3.2. Characterization

The set of parameters BET surface area, the volume of micropore and the total volume of pores were derived from the N_2_ adsorption-desorption isotherms, at −196 °C using a Micromeritics equipment (Gemini V). All samples were outgassed at 150 °C for 2 h prior to the analysis. The specific surface area was derived by the Brunauer–Emmet–Teller equation (S_BET_), in according to the multipoint analysis described by Rouquerol and coworkers [80] and Thommes and coworkers [56]. Total pore volume was reached as the amount adsorbed at relative pressure P/P_o_ > 0.99 (V_0.99_), while micropore volume V_DR_ was determined by the Dubinin–Radushkevich method [80]. Mesopore volume V_meso_ was estimated by the difference between V_0.99_ and V_DR_. For the sake of comparison, the density functional theory (DFT) [57,58] method was also used to evaluate the porosimetry properties. Structural characterization was carried out by X-ray diffraction (XRD) in a diffractometer (model XRD-6000, Shimadzu, Kyoto, Japan) with Cu-Kα_1_ radiation (λ = 1.54056 Å) and graphite monochromator. Measurements were carried out over an angular range of 5° ≤ 2θ ≤ 80° with a scanning step of 0.02° and a fixed counting time of 10 s. Divergence, scattered, and receiving radiation slits were 1°, 1°, and 0.2 mm, respectively. Chemical bonds were investigated by infrared spectroscopy (FT-IR) using a Fourier transform spectrometer model Digilab Excalibur (FTS 3100 HE series, Hopkinton, MA, USA). Samples were diluted in KBr in a ratio of 1:100. Measurements were carried out in the range of 4000–400 cm^−1^, with a resolution of 8 cm^−1^ and 100 scans. Surface pH pH_PZC_ were obtained by the drift-pH method [25] following the change of pH in aqueous suspension with respect to time until a constant pH is observed. Microstructural analysis of the composite and the amorphous carbon matrix was performed by scanning electron microscopy (SEM) using a microscope Carls Zeiss EVO LS15 model operating at 30 kV on samples after coating with a thin layer of gold. Elemental analysis was conducted in a Vario EI elemental analyzer to determine the mass fractions of carbon, hydrogen, and nitrogen (CHN) of the samples. Oxygen content was determined by the difference between 100% and C, H, and N fractions (%) using the remaining fraction of Zn and O obtained from thermogravimetric analysis of the samples. Thermogravimetric analysis (TGA) under O_2_ flow was performed to obtain the residual mass of samples composed by zinc and oxygen. Diffuse reflectance spectra of samples (UV-vis/DRS) were recorded in air at ca. 300 K in the wavelength range 250–800 nm, using a Shimadzu UV-2401 PC spectrophotometer (Kyoto, Japan) with BaSO_4_ as the reference material. Quantification of Zn was performed in an atomic absorption (AA) spectrophotometer from Perkin Elmer (model Analyst 400, series 201S3090705, Waltham, MA, USA). The quantification was performed by using a calibration curve with five different concentrations from a standard from FisherScientific. The wavelength used for the analysos of Zn was 213.9 nm. Analysis was done by triplicate and the reproducibility of results was better than 99%.

### 3.3. Adsorption in the Dark and Photocatalytic Tests

Preliminary studies of adsorption of azo-dye basic blue 41 called BB41 in the dark were performed to guarantee the correct interpretation of the degradation results obtained under irradiation. The experimental conditions are described as follows. The kinetics of the BB41 adsorption in the dark was studied at 25 °C, with an initial concentration of 12.5 ppm (2.59 × 10^−5^ mol L^−1^), in a volume of 1 L (25.9 μmol BB41) and sample weights of 100 mg. The loading of samples used in the present work was constant, ca. 0.1 g L^−1^. A detailed analysis for the kinetics of adsorption was obtained by comparing the pseudo-first order [81], the pseudo-second order [82], and the intraparticle diffusion [83,84] kinetic models. The Table S1 (Supplementary Materials) contains a summary of the kinetic expressions and parameters used in the present study of adsorption in the dark. It is important to point out that for the preparation of the solutions of BB41 with the initial concentration indicated above, the weight used of the dye was corrected using a factor of 2.375 to achieve a nominal purity of 95%. The photocatalytic activity of samples was studied following the kinetics of BB41 degradation as a function of irradiation time. 12.5 ppm BB41 initial concentration, 1 L of volume solution, 100 mg weight, and a loading ca. 0.1 g L^−1^ were used in all the tests. An open-to-air batch photoreactor [64,85] was employed consisting of a cylindrical flask made of Pyrex (ca. 2000 mL) with a bottom optical window of ca. 10 cm diameter. A solar simulator box equipped with a Xe-lamp (400 W) emitting the solar spectrum was used as source of irradiation. The total photon flux was measured with a pyranometer yielding ca. 3.2 × 10^19^ photons m^−2^ s^−1^. The UV and visible light components were estimated by integration of the radiation spectrum [64] giving 0.19 × 10^19^ photons m^−2^ s^−1^ and 3.01 × 10^19^ photons m^−2^ s^−1^ for the UV and visible light components, respectively. It means only ca. 6% of UV component. Aliquots were taken at different times and centrifuged firstly at 2000 rpm for 5 min following 3500 rpm for 5 min to separate the solid from aqueous phase. Then, BB41 concentration was analyzed by UV-visible spectroscopy (Perkin Elmer, UV-Vis Lambda 365) at 609 nm. Before irradiation, 60 min was considered as the minima time required to achieve equilibrium of adsorption. Accordingly, the data obtained from the kinetics of BB41 degradation were normalized to its concentration after 60 min of adsorption in the dark. This normalization permits the correct estimation of the photoactivity of the materials. The photodegradation of BB41 was analyzed using a First-order reaction-rate mechanism to estimate the apparent rate-constant k_app_ from the linear regression of the kinetic data, by using Equation (1) where C_o_ and C_t_ represent the BB41 initial concentration and the concentration at the time t, respectively, as follows:

Ln(C_o_/C_t_) = k_app_ × t (1)

Results of BB41 adsorption and photodegradation obtained on the composites were compared against those obtained with the amorphous carbon and the commercial ZnO. Direct photolysis experiments were also conducted as blank test, and all the photocatalytic tests were done by duplicate and the deviation was lower than a 3%.

## 4. Conclusions

The synthesis of complex hybrid composite based on both ZnO and/or metallic Zn dispersed in an amorphous-carbon matrix by modified Pechini route accomplished partial pyrolysis method is fully operational. Both techniques, X-ray diffraction and FTIR, showed that metallic zinc is disperse in amorphous carbon. Infrared spectra of the composites exhibited bond vibrations associated to the surface functional groups characteristic of amorphous carbon. Only the C/ZnO/Zn composite exhibited characteristics diffraction lines and vibrational bands related to the bond vibrations of zinc oxide. Despite of the functional groups (polyesters) in the surface of the amorphous carbon are responsible to hinder the adsorption of BB41 molecules, the presence of the amorphous carbon enhanced the photocatalytic activity of the ZnO by a factor of ca. 2.4, and this enhancement in photoactivity, as well as the stability of the C/ZnO/Zn and C/Zn composites, was correlated to a protector effect by the carbon layers from lixiviation phenomena. Additional experiments showed a high photocatalytic activity and stability to leaching of Zn after four consecutive photocatalytic runs using C/ZnO/Zn composite. Thus, the present materials have a great potential to be used for the remediation of polluted water.

## Figures and Tables

**Table 1:** Assignment of the FTIR absorption bands observed in the present samples.

Assignments	Wavenumber (cm^−1^)	References
*ν_sim_* (OH) water	3546–3373	[46,47]
*ν_sim_* (C–H)	2943	[27,28]
*ν_sim_* (C=O) ester	1748–1736	[29,49,50]
δ O–H, δ_ass_ C–H	1607–1403	[28,48]
*ν* (C–O) ester, δ_sim_ C–H	1383–1037	[29,49,50]
*ν_sim_* (C–C)	963–753	[27,28]
Vibrations Zn–O	672–563	[45,51,52]

**Table 2:** List of the textural properties of the samples.

Sample	S_BET_^a^ (m^2^ g^−1^)	V_DR_^b^ (cm^3^ g^−1^)	V_0.99_^c^ (cm^3^ g^−1^)	V_meso_^d^ (cm^3^ g^−1^)	V_<1 nm-DFT_^e^ (cm^3^ g^−1^)
ZnO	13	0.001	0.048	0.047	0.001
C-amorphous	196	0.230	0.298	0.066	0.094
C/Zn	125	0.050	0.190	0.140	0.013
C/ZnO/Zn	75	0.022	0.114	0.092	0.006

^a^ BET specific surface area. ^b^ Micropore volume (V_DR_) determined by the Dubinin–Radushkevich method. ^c^ Total pore volume (V_0.99_) obtained as the N_2_ amount adsorbed at relative pressure P/P_o_ > 0.99. ^d^ Mesopore volume (V_meso_) estimated by the difference between V_0.99_ and V_DR_. ^e^ Volume of pores with diameters lower than 1 nm estimated by density functional theory (DFT).

**Table 3:** List of kinetic results for the BB41 adsorption in the dark and pH_PZC_ of the samples.

Sample	BB41_ads-eq_^a^ (μmol)	k_1_^b^ (min^−1^)	R_k1_^c^	k_2_^d^ (μmol^−1^ min^−1^)	R_k2_^e^	k_p_^f^ (μmol min^−0.5^)	C ^g^ (μmol)	R_kp_^h^	pH_PZC_^i^
ZnO	1.95	0.049	0.911	0.216	0.951	0.056	1.51	0.973	8.9
C-amorphous	0.54	0.048	0.958	1.491	0.907	0.031	0.30	0.714	5.4
C/Zn	0.87	0.037	0.946	0.746	0.940	0.025	0.68	0.704	6.6
C/ZnO/Zn	1.46	0.060	0.957	0.686	0.949	0.047	1.10	0.737	7.9

^a^ BB41 adsorbed after 60 min (pseudo-equilibrium conditions). ^b^ Kinetic constant for the BB41 adsorption according to the pseudo-first order. ^c^ Regression factor for k_1_. ^d^ Kinetic constant according to the pseudo-second order. ^e^ Regression factor for k_2_. ^f^ Kinetic constant according to the IPD model. ^g^ C is the layer thickness of molecules close to the surface of the adsorbent according to the IPD model. ^h^ Regression factor for the IPD model. ^i^ Surface pH estimated by the drift-pH method according to Ref. [25].

**Table 4:** List of kinetic parameters for the BB41 adsorption in the dark and photocatalytic degradation under solar irradiation.

Sample	BB41_ads-eq_^a^ (μmol)	k_app_ × 10^−3^ (min^−1^) ^b^	R_kapp_^c^	Φ_rel-ZnO_^d^	BB41_Conv-3h_^e^ (%)	BB41_Conv-__6h_^f^ (%)
Photolysis	-	0.5	0.960	0.07	6	7
ZnO	1.95	7.3	0.991	1.0	69	85
C-amorphous	0.54	1.6	0.958	0.2	19	25
C/Zn	0.87	9.2	0.996	1.3	78	94
C/ZnO/Zn	1.46	17.2	0.996	2.4	96	100

^a^ BB41 adsorbed after 60 min (pseudo-equilibrium conditions). ^b^ First-order apparent rate-constant. ^c^ Regression factor for k_app_. ^d^ Φ_rel-ZnO_ is the photocatalytic activity relative to ZnO (k_app-i_/k_app-ZnO_). ^e^ BB41 fraction converted after 3h irradiation. ^f^ BB41 fraction converted after 6h irradiation.

**Table 5:** Summary of the photocatalysts´ composition.

Sample	C ^a^ (%)	H ^a^ (%)	O ^a^ (%)	Zn + O ^b^ (%)
ZnO	-	-	-	100
C-amorphous	93	0.5	6.5	--
C/Zn	59	0.3	3.7	37
C/ZnO/Zn	52	0.3	3.7	44

^a^ Determined by elemental analysis. ^b^ Determined by thermogravimetric analysis.

**Table 6:** Summary of the kinetics results obtained for the consecutive catalytic photodegradations of BB41 using C/ZnO/Zn and values obtained for the leaching of Zn during after the catalytic runs.

Photocatalytic Run	k_app_ × 10^−3^ (min^−1^) ^a^	R^2 b^	Δk_app_^c^ (%)	Zn ^d^ (g L^−1^)	Leached Zn ^e^ (%)
1	17.2	0.996	0	<LOD ^f^	0
2	16.8	0.992	2	0.0005	1.4
3	15.9	0.988	7	0.0008	2.2
4	14.7	0.990	14	0.0012	3.4

^a^ First-order apparent rate-constant. ^b^ Regression factor for k_app_. ^c^ Loss in activity along the consecutive photocatalytic runs. ^d^ Zn determined in aqueous phase by atomic absorption spectrometry. ^e^ Zn leached after each photocatalytic run with respect to the initial composition of Zn in the catalysts: 0.035 g L^−1^. ^f^ Below limit of detection (<LOD).

## Supplementary Materials

The Supplementary Materials are available online.
