# Supplementary material for: Hybrid Material Based on an Amorphous-Carbon Matrix and ZnO/Zn for the Solar Photocatalytic Degradation of Basic Blue 41
Source: Molecules. 2019 Dec 26;25(1):96. doi: 10.3390/molecules25010096 (PMC6983089; doi:10.3390/molecules25010096)
Supplement: Supplementary file 1 [file molecules-25-00096-s001.zip › molecules-651507-SI.pdf]

**Supplementary Material**

# Hybrid material based on an amorphous-carbon matrix and ZnO/Zn for the solar photocatalytic degradation of basic blue 41

Silvania Lanfredi[1,*], Marcos A. L. Nobre[2], Po S. Poon[3], Juan Matos[3,4,*]

[1]Department of Chemistry and Biochemistry, São Paulo State University (Unesp), School of Technology and Sciences, Laboratory of Composites and Ceramics Functional, Presidente Prudente, Brazil.

[2]Department of Physics, São Paulo State University (Unesp), School of Technology and Sciences, Presidente Prudente, Brazil.

[3]Technological Development Unit (UDT), University of Concepcion, Concepcion, Chile.

[4]Millennium Nuclei on Catalytic Processes towards Sustainable Chemistry (CSC), Chile.

*Corresponding authors. Emails: jmatoslale@gmail.com (J. Matos); silvania.lanfredi@unesp.br (S. Lanfredi)

**Table S1.** Summary of kinetics model for the analysis of the kinetic data of BB41 adsorption.

| **Kinetic model** | **Equation** | **Reference** |
|---|---|---|
| **Pseudo-first-order** | $\log(BB41_{ads-eq} - BB41_{ads-t}) = \log(BB41_{ads-eq}) - (k_1/2.303)\ t$ | 81 |
| **Pseudo-second-order** | $(1/\ BB41_{ads-eq} - BB41_{ads-t}) = (1/BB41_{ads-eq}) + k_2\ t$ | 82 |
| **Intraparticle diffusion** | $BB41_{ads-t} = C + k_p\ t^{1/2}$ | 83,84 |

The pseudo-first-order model showed in Eq. (1) where $k_1$ is the pseudo-first-order rate constant ($min^{-1}$) for the adsorption. $BB41_{ads-t}$ is the amount of BB41 adsorbed (in μmol) at time t (min) and $BB41_{ads-eq}$ is the amount adsorbed at equilibrium (in μmol).

$$dBB41_{ads}/dt = k_1(BB41_{ads-eq} - BB41_{ads-t}) \qquad (1)$$

The integration of Eq. (1) at the initial conditions ($n_{ads} = 0$ at $t = 0$) yields the Eq. (2):

$$\log(BB41_{ads-eq} - BB41_{ads-t}) = \log(BB41_{ads-eq}) - (k_1/2.303)\ t \qquad (2)$$

In addition, a pseudo-second-order equation may be expressed by Eq. (3):

$$dBB41_{ads}/dt = k_2(BB41_{ads-eq} - BB41_{ads-t})^2 \qquad (3)$$

Where $k_2$ is the pseudo-second-order rate constant ($\mu mol^{-1}.min^{-1}$) for the adsorption. Applying the initial conditions, Eq. (3) can be integrated to obtain:

$$(1/BB41_{ads-eq} - BB41_{ads-t}) = (1/BB41_{ads-eq}) + k_2.t \qquad (4)$$

The influence of the intraparticle diffusion phenomena upon the adsorption capacity of BB41 was verified keeping in mind that the fractional approach to equilibrium change is done according to a function of $(D_t/r^2)^{1/2}$, where r is the radius of adsorbent particle, and D is the effective diffusivity of solute within the particle. Thus, the initial rate according to the intraparticle diffusion model (IPD) is obtained from the liner regression of the curve $BB41_{ads-t} = f(t^{1/2})$, expressed by the Eq. (5), where $k_p$ is the IPD rate constant ($\mu mol\ min^{-0.5}$), and C is a constant (μmol) attributed to the extension of the boundary layer thickness.

$$BB41_{ads-t} = C + k_p\ t^{1/2} \qquad (5)$$

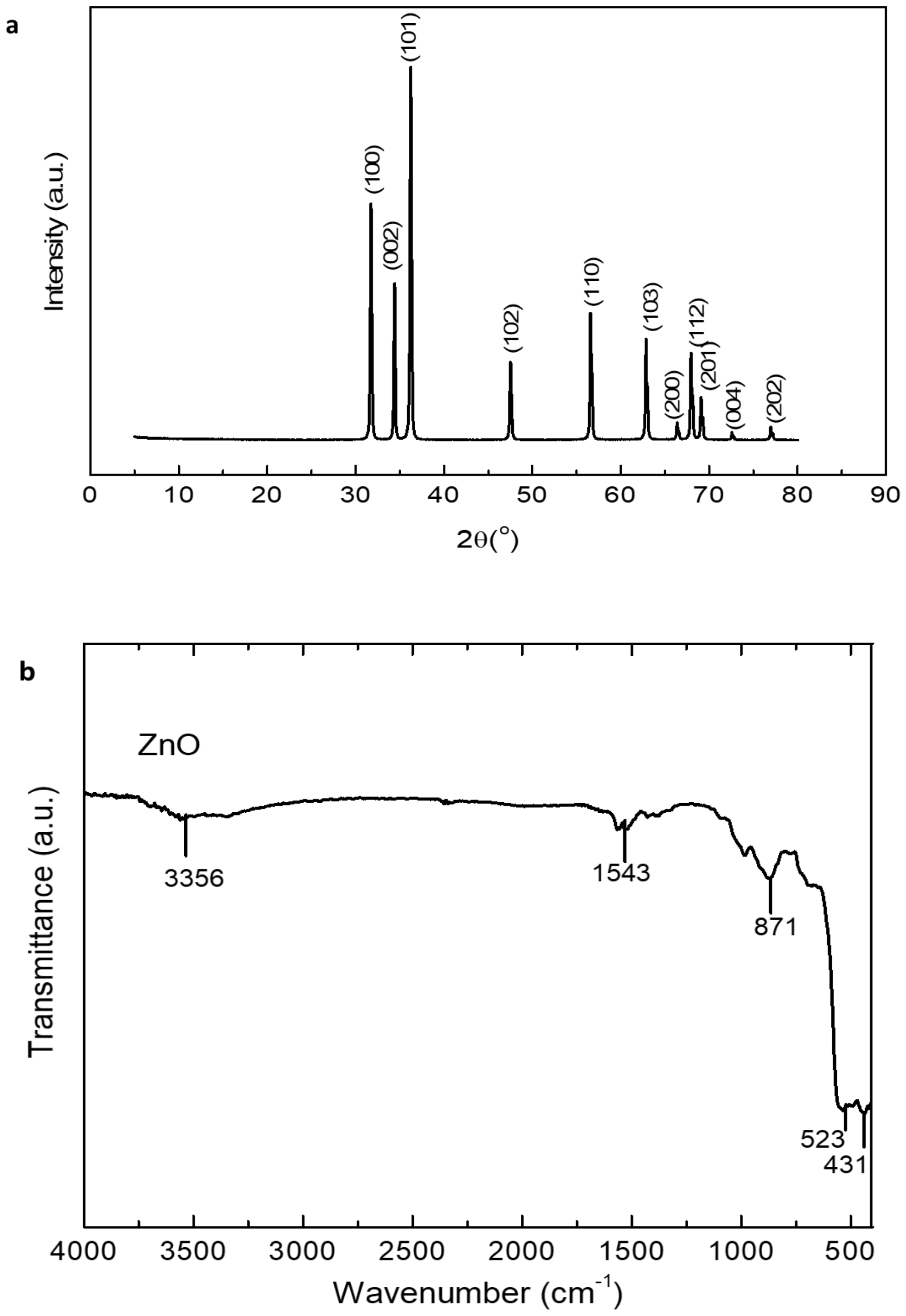


**Figure S1**. Characterization of commercial ZnO. (a): XRD pattern. (b): FTIR spectra.

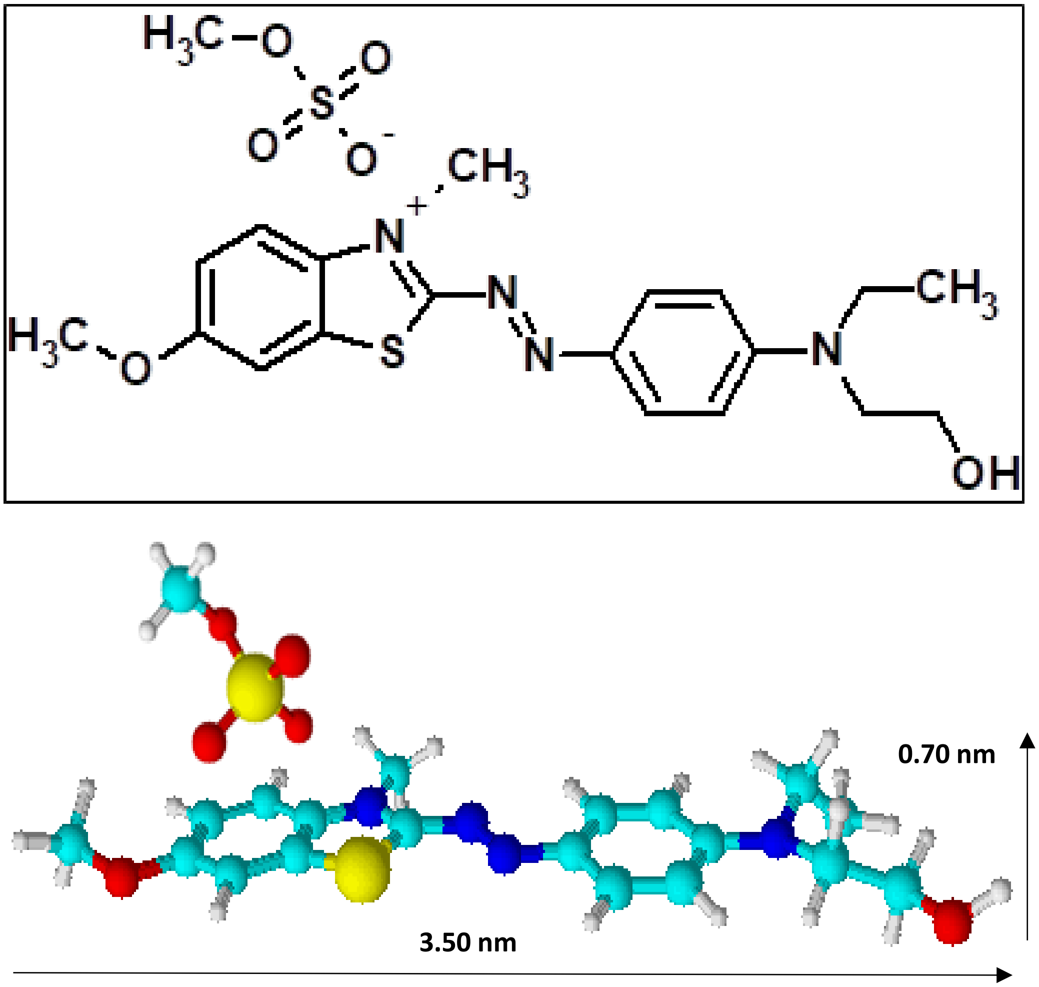


**Figure S2.** Molecular structure of basic blue 41.

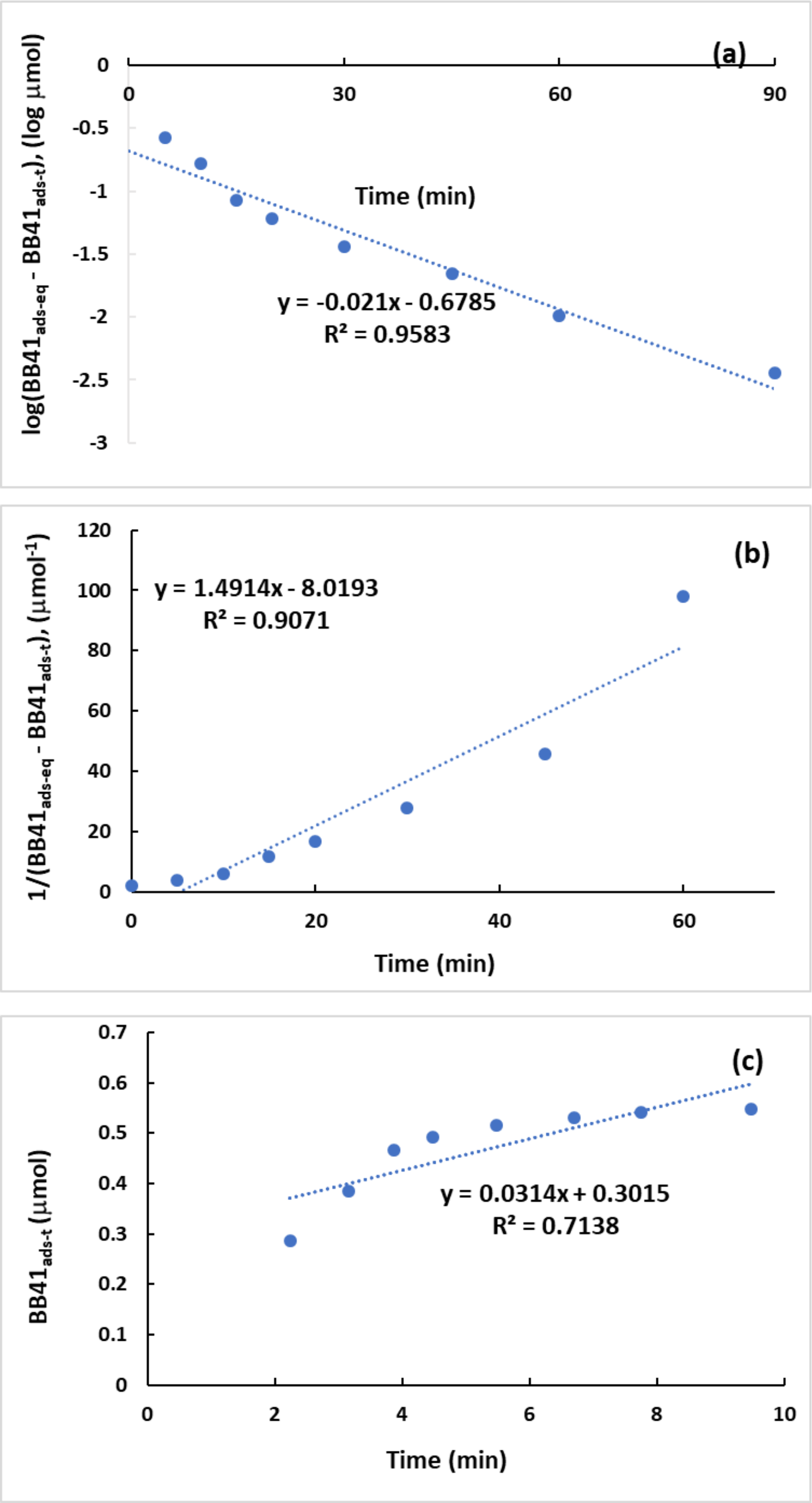


**Figure S3.** Kinetic treatment of the data of BB41 adsorption on C-amorphous. (a): Pseudo-first order. (b): Pseudo-second order. (c): IPD model.

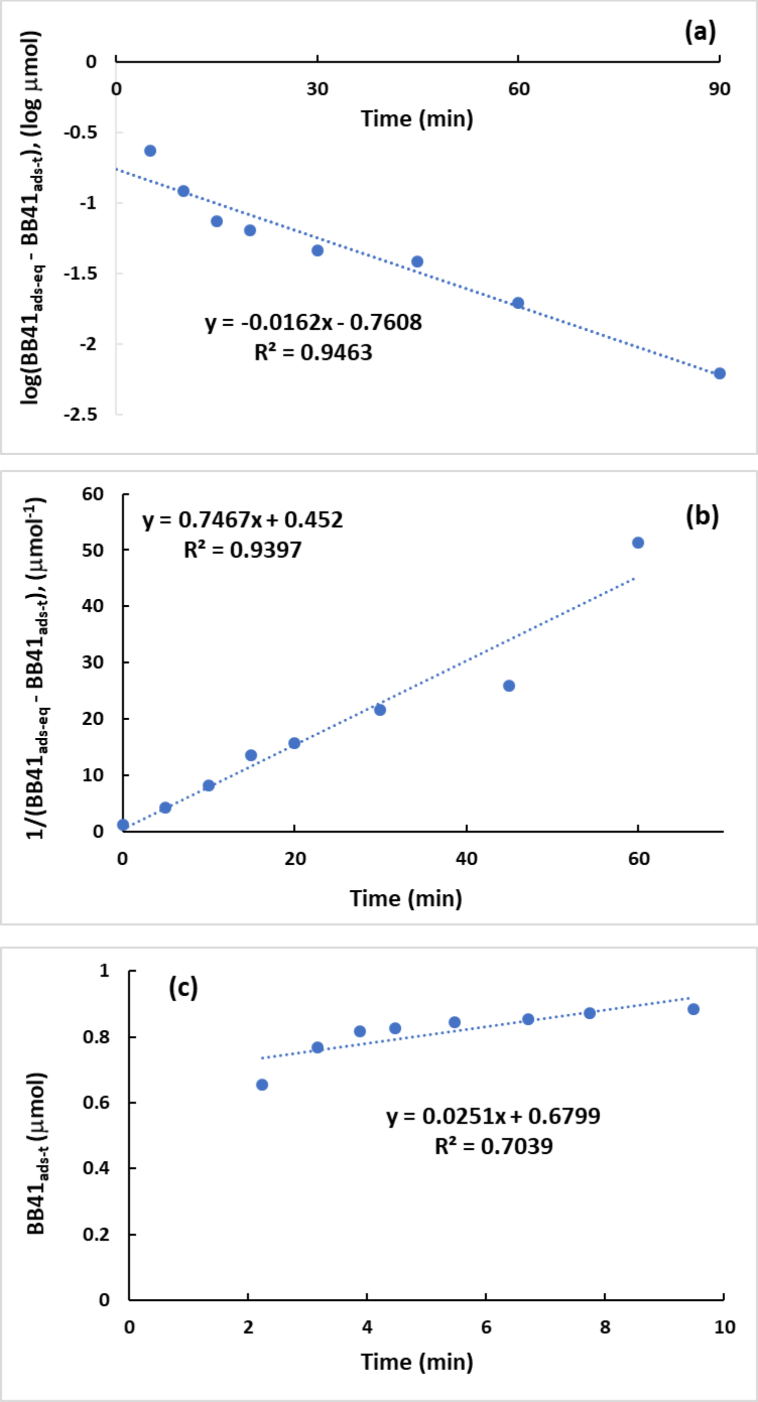


**Figure S4.** Kinetic treatment of the data of BB41 adsorption on C/Zn. (a): Pseudo-first order. (b): Pseudo-second order. (c): IPD model.

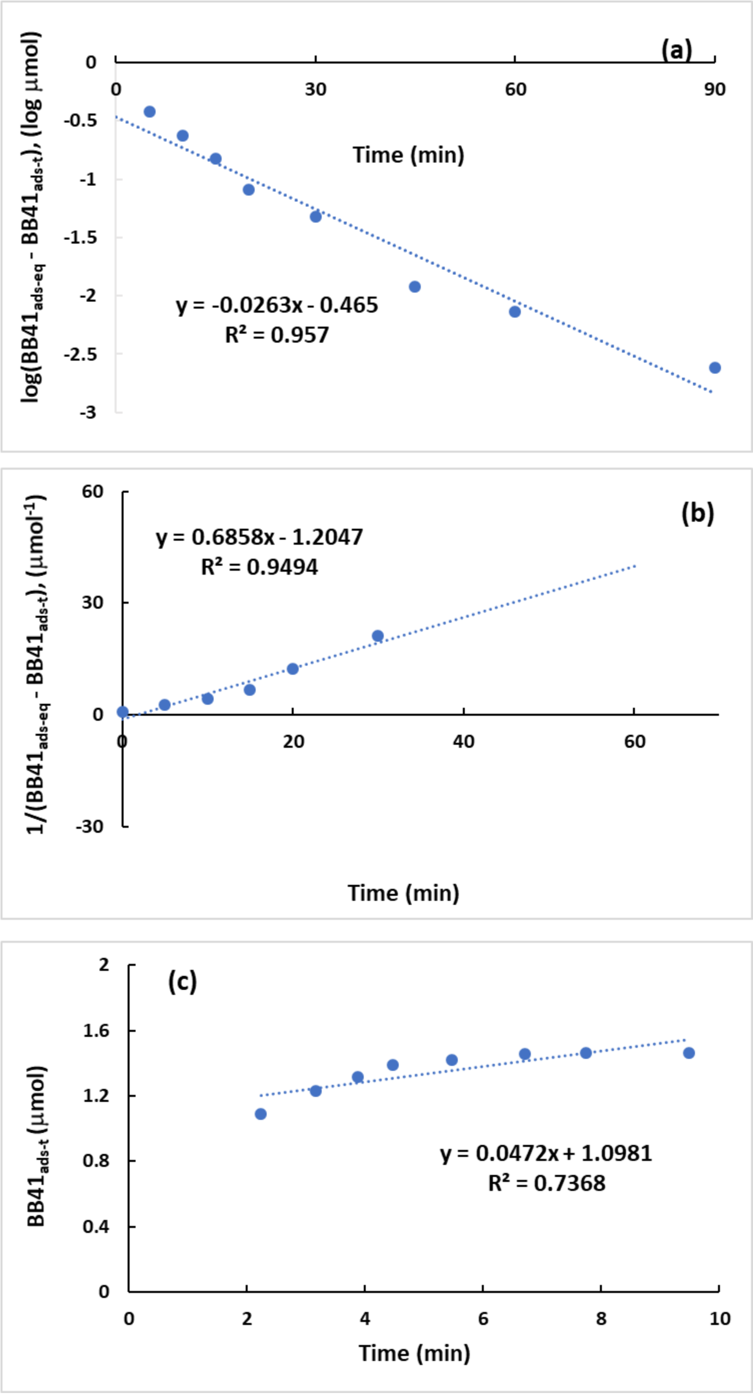


**Figure S5.** Kinetic treatment of the data of BB41 adsorption on C/ZnO/Zn. (a): Pseudo-first order. (b): Pseudo-second order. (c): IPD model.

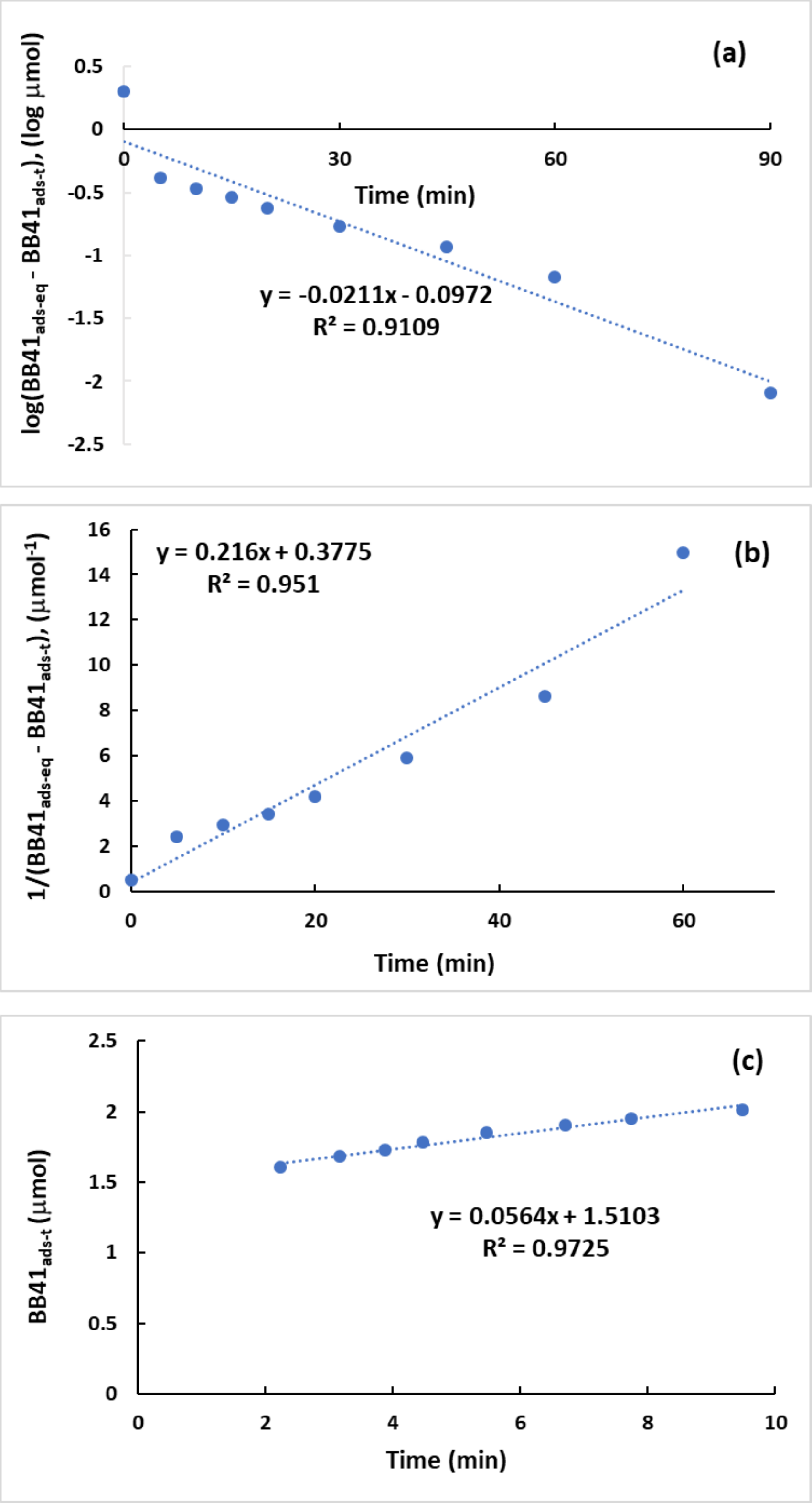


**Figure S6.** Kinetic treatment of the data of BB41 adsorption on ZnO. (a): Pseudo-first order. (b): Pseudo-second order. (c): IPD model.
